# Incidence of Intestinal Infectious Diseases due to Protozoa and Bacteria in Mexico: Analysis of National Surveillance Records from 2003 to 2012

**DOI:** 10.1155/2018/2893012

**Published:** 2018-07-15

**Authors:** Daniel Diaz, Aldo M. Vazquez-Polanco, Jesus Argueta-Donohue, Christopher R. Stephens, Francisco Jimenez-Trejo, Santa E. Ceballos-Liceaga, Natalia Mantilla-Beniers

**Affiliations:** ^1^Centro de Ciencias de la Complejidad (C3), Universidad Nacional Autónoma de México, Ciudad de México 04510, Mexico; ^2^Facultad de Medicina Veterinaria y Zootecnia, Universidad Autónoma de Sinaloa, Culicán de Rosales 82260, SIN, Mexico; ^3^Instituto Nacional de Psiquiatría “Ramón de la Fuente Muñiz”, Ciudad de México 14370, Mexico; ^4^Instituto de Ciencias Nucleares, Universidad Nacional Autónoma de México, Ciudad de México 04510, Mexico; ^5^Instituto Nacional de Pediatría, Ciudad de México 04530, Mexico; ^6^Dirección General de Epidemiología, Ciudad de México 01480, Mexico; ^7^Departamento de Matemáticas, Facultad de Ciencias, Universidad Nacional Autónoma de México, Ciudad de México 04510, Mexico

## Abstract

**Background:**

According to national epidemiological surveillance records, in Mexico six intestinal infectious diseases (IID) are among the top infectious communicable diseases. However, their incidence, relative importance, and spatial patterns have not been studied in detail.

**Aims:**

We examine the epidemiology of IID due to bacteria and protozoa to identify which diseases are most important at two spatial scales, what is their integrated importance locally, and how their incidence correlates with Human Development Index (HDI).

**Methods:**

We retrieved yearly number of new cases of eight IID from the national epidemiological morbidity report from 2003 to 2012 at the national level, by state, and to assess such information at a higher spatial resolution we included the municipalities for Mexico City. However, no comparisons were made to other municipalities due to unavailability of data. We compared incidence, obtained the disease-specific relative importance, and inspected spatial patterns for the integrated incidence. Finally, we tested whether HDI is correlated with incidence.

**Results:**

We found that, except for two diseases, the relative importance of the other six IID contrasted not only between the national level and Mexico City, but also among states and municipalities in Mexico City. Besides, at both scales the distribution of the incidence showed disease-specific spatial patterns. Finally, there was a lack of consistent correlation between HDI and individual IID at both scales.

**Conclusion:**

Our results emphasize the need for local disease-focused selective models for control and prevention of IID. The maps displaying our analyses of epidemiological similarities may be used in orienting such effort.

## 1. Introduction

Diarrhoeal diseases were the second most common acute disorders in 2013, causing 2.7 billion cases worldwide [1]. Even though from 1990 to 2013 there was a 7.9% reduction in their incidence [1], they are among the most important communicable diseases globally [2]. Indeed, by 2013 diarrhoeal diseases were ranked 4^th^ among all causes of death worldwide [3]. Furthermore, diarrhoeal diseases cause great negative socioeconomic impact: by 2013 they were at the 4^th^ place among the top 50 causes of Years of Life Lost (YLL) worldwide [1]. Even though these diseases do not discriminate between age and social status, the most vulnerable groups include children and elderly people, especially those living in low-income countries [4].

Although there are several causes of diarrhoea (infections, malnutrition, and toxins found in contaminated food or water), intestinal infectious diseases (IID) are one of the most important causes. IID are caused by a broad variety of infectious agents: protozoa, bacteria, helminths, and viruses. These pathogens are mostly transmitted via the faecal-oral route, through contaminated food and water, and occasionally from person to person [2, 5] and are well known for the symptoms they share: inflammatory gastrointestinal processes, diarrhoea, dehydration, malnutrition, nausea, vomit, abdominal pain, and fever [4]. Because of the ensuing dehydration, diarrhoeal diseases are particularly dangerous for children [6, 7]: they constitute the third cause of death in the pediatric population from 1 to 59 months of age [3] and in 2013 were a leading cause of death in children aged 5-9 years [8].

Lack of adequate sanitation [9], deficient hygiene [10], and inefficient water treatment [11] are often cited as the main causes of increased IID morbimortality in middle- and low-income countries. Sociodemographic indicators have been shown to be related to differential burden of diarrhoeal diseases [12]. In high-income countries IID are not among the top 10 causes of YLL, where they invariably figure in developing countries [3]; thus, the burden of these diseases in high-income countries contrasts starkly with that experienced in middle- and low-income countries [11].

In Mexico, six IID consistently appeared from 2011 to 2014 among the top twenty communicable diseases [13–15], which already marks them as an important health problem. However, few studies have comprehensively described the epidemiology of IID in Mexico. A report published in 2012 by the national Dirección General de Epidemiología [16] presents 30 years (1980-2010) worth of morbimortality IID data by age group, state, and gender but does not discuss disease-specific incidence and mortality. In contrast, Hernandez et al. [17] provide national, annual disease-specific trends of IID cases, while failing to examine state-level incidence. In turn, Cifuentes et al. [18] report the temporal changes in the spatial distribution of mortality due to the diarrhoeal diseases focusing on cholera, and Flisser et al. [19] analyze survey data for cholera and rotavirus from 1995 to 2000. Other studies assess the prevalence or incidence of intestinal parasitosis [20–22] or endoparasitic infections [23–26] that cause diarrhoeal diseases in different regions of Mexico or the association between incidence of diarrhoeal diseases and age, socioeconomic status, and other risk factors [27–30].

This paper seeks to provide a clearer picture of IID by detailing which types are most important in a given geographic location, what is their incidence, and how it is correlated with Human Development Index (HDI), a proxy of socioeconomic status. Mexico is ranked as having high HDI, with 8 of its 32 states deemed to have very high HDI, while half of its population lives in states with low or medium HDI [31]. Socioeconomic factors have been found in the past to have a negative association to incidence of IID and diarrhoeal disease in general [6, 32]. Thus, regions with geographic proximity and common socioeconomic determinants are expected to present similar epidemiological patterns [32]. We tested this hypothesis at two geographic levels: nationally, by state, and in Mexico City, by municipality. In the latter almost all municipalities are considered to have very high HDI, thus sharing socioeconomic features and geography, while at the national level there is considerable diversity between states.

## 2. Methods

### 2.1. Epidemiological Importance of IID

It is standard practice to select diseases based on both public health importance and data availability [33]. Following this practice, we chose intestinal diseases of compulsory notification in Mexico, six of which appear among the twenty most important communicable diseases from 2011 to 2014. Our analyses allow us to describe broad incidence patterns and identify national priorities for control. To attain these objectives we analyzed national data from all 32 states of Mexico. We also inspected the epidemiological landscape of gastrointestinal infections at higher spatial resolution by studying their incidence in the 16 municipalities of Mexico City. Only this city was included at the municipality level due to unavailability of complete data sets for others states.

The number of new cases of IID was retrieved from the yearly morbidity report of the Dirección General de Epidemiología in Mexico [34]. Although cases are recorded weekly, we analyzed yearly counts from 2003 to 2012 for the 32 states of Mexico and the 16 municipalities of Mexico City. In the next section, we detail how data are collected.

### 2.2. Data Collection Protocols

Epidemiological surveillance in Mexico is based on a standardized procedure for weekly notification of new cases [34]. Disease notification concentrates data at four levels: (1) Clinic/Hospital, (2) Sanitary Jurisdiction, (3) State, and (4) Country. A paper form (SUIVE-1) is filled in at the first level and is captured, analyzed, and verified in the SUAVE (Sistema Único Automatizado para la Vigilancia Epidemiológica) software. Thus, the standardized procedure for notification of new cases involves (1) completion of SUIVE format, (2) data capture, compilation, and analyses using SUAVE software, and (3) data validation according to standard international guidelines (ISO 9000:2005). As a result, the weekly report of new cases is built and updated systematically with the participation of a vast network of epidemiologists across the country and includes the entire National Health System network of public health institutions, as well as private clinics and hospitals.

### 2.3. Disease Selection and Classification

In Mexico, 142 diseases classified in 16 groups are of compulsory notification [34] and 14 IID form one of such groups. We report here our analyses of eight gastrointestinal disease categories, selected on the following grounds: cholera caused no new cases during the period studied; four diseases of the remaining 13 are compacted into two: paratyphoid fever and other salmonelloses are grouped together, as are intestinal amoebiasis and amoebic liver abscesses. Since there are no records of ascariasis, other helminthiases, or enterobiasis for the municipalities of Mexico City, we exclude these diseases and keep only the eight IID for which records are complete. According to the definitions given in Chapter I “certain infectious and parasitic diseases” of the International Statistical Classification of Diseases and Related Health Problems (ICD-10) Version: 2016 [35], the eight gastrointestinal diseases included in our study are grouped under the category* intestinal infectious diseases* (codes A00-B99). Their codes, etiological agents, and acronyms used for each particular disease are summarized in Table 1.

### 2.4. Estimation of Incidence

Due to the discrete nature of the data, we used the median as a descriptive and comparative measure of central tendency for the incidence (cases per 100 000 inhabitants) during 2003-2012. Population estimates were obtained at the national, state, and municipality level from the Instituto Nacional de Estadística y Geografía in Mexico. We constructed a 95% confidence interval (CI) for the median as proposed by Zar [36]; for this, we considered a binomial distribution with p = 0.5 and calculated according to

(1) PXi≤median≤Xj≥1- α,where  i=Cα2,n+1  and  j=n−Cα2,n

It is worth remembering that the CI of the median is not expected to be symmetrical.

To compare which median incidences during 2003-2012 at the state or municipal level were above or below their corresponding reference point (national or Mexico City median, respectively) we used mean rank comparison with a Kruskal-Wallis test as reported elsewhere [37]. Then, we used a Dunn's test corrected for multiple comparisons whenever the null hypothesis was rejected; i.e., all localities (states or municipalities) had the same median incidence. We selected this nonparametric test considering the nonnormal data distributions and incidence variance heterogeneity assessed as reported elsewhere [38]. These analyses were done in Prism 7 (GraphPad Software, Inc.); a value of* p* < 0.05 was considered significant.

### 2.5. Spatial Distribution of Disease

We studied spatial patterns of IID incidence for the states and municipalities. To present our results we first drew a map of disease incidence for individual infections with SAS 9.4 (SAS Institute), coloring localities by percentile incidence. Next, we obtained an integrated epidemiological landscape of IID incidence through (1) Z-scores of the median incidence for each disease, (2) heatmaps and dendrograms built with cluster analysis of the Z-scores of the medians at each level, and (3) maps summarizing cluster analyses results. Heatmaps and figures were constructed with Prism 7 and multivariate analyses were done in IBM SPSS 21 (IBM Corp.).

### 2.6. Association of Incidence and Socioeconomic Indicators

Various measures of development have been used to evaluate their association with disease mortality, incidence, and/or prevalence [6, 39, 40]. We used the Human Development Index (HDI) [41] as a summary measure of socioeconomic status and studied whether it has a significant linear relation with disease incidence. We obtained HDI values at the state and municipal level [31]. For this, we used the two-tailed Spearman's rank correlation analysis in Prism 7.

## 3. Results

### 3.1. Intestinal Infectious Diseases in Mexico: A Major Health Issue

**Figure 1:**
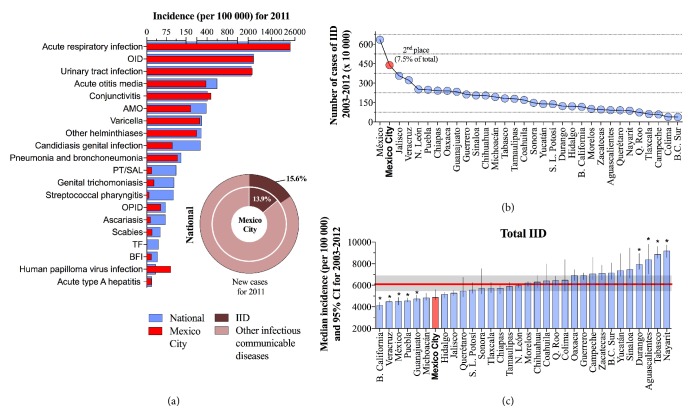
(a) Rate of incidence for 2011 of the top 20 infectious communicable diseases at the national level and for Mexico City, (b) state contribution to the national incidence of gastrointestinal disease during 2003-2012, and (c) decade median incidence of the total cases of IID by state.

According to the national epidemiological surveillance records [15], in Mexico recently six IID lied among the top 20 most important communicable diseases. Hence, to put into context the importance of IID as a public health problem for the population of Mexico, in Figure 1 we present as a reference the incidence for 2011 of the group of infectious communicable diseases that caused the highest morbidity. Thus, as shown in Figure 1(a) six IID were among the top 20 communicable diseases during 2011 at both spatial scales. They made up 13.9% of the top 20 in Mexico City and 15.6% at the national level. At both scales, the most important category of IID was OID in 2011, which ranked second among the top 20 communicable diseases. In fact, the striking importance of OID in the context of infectious diseases became even more evident when we studied the fraction they represented of all intestinal infections during the decade: 83.5 and 90.6% nationally and in Mexico City, respectively.

During the decade starting in 2003, the eight IID that we evaluated caused 58.29 million cases at the national level, 4.38 million (7.5%) of which were located in Mexico City, only behind the 10.9% of reports recorded in Estado de México (Figure 1(b)). However, total IID incidence (cases per 100 000) observed in Mexico City (4878, 95% CI: 4656-5598) was below the national median of 6089 (95% CI: 5461-6897), only above Baja California, Veracruz, Estado de México, Puebla, Guanajuato and Michoacán (Figure 1(c)).

### 3.2. Amoebiasis, a Key Contributor

**Figure 2:**
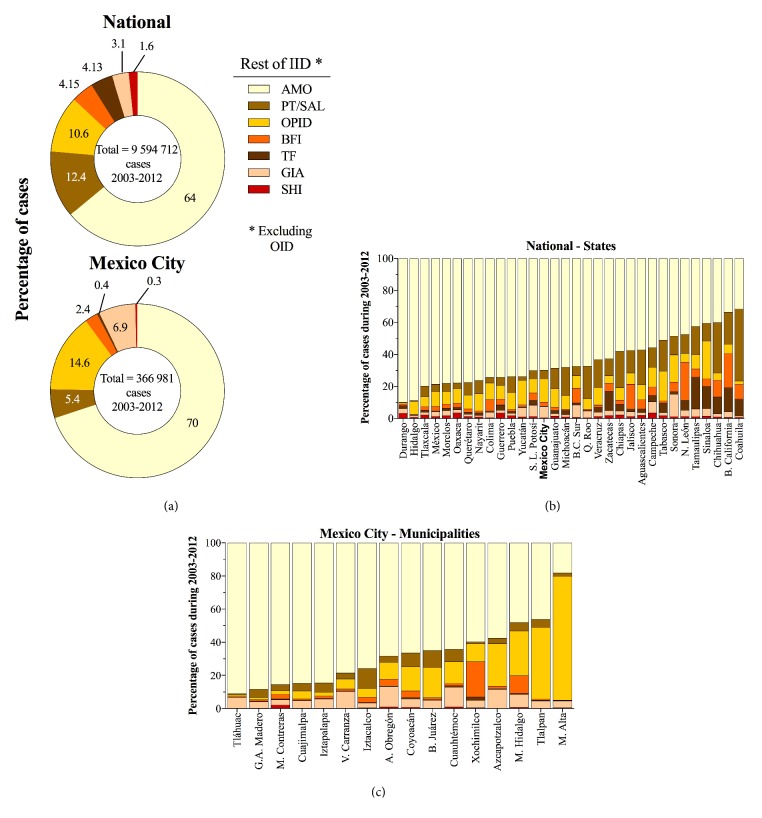
(a) Percentage of cases of the rest of IID, excluding OID, at the national level and for Mexico City, and (b) relative contribution of each class of intestinal infection, excluding OID, to the incidence of gastrointestinal incidence in the states at the national level and in the municipalities of Mexico City.

The relative contribution of IID outside the OID class between 2003 and 2012 is shown in Figure 2(a); AMO was the most important contributor, causing 64 and 70% of cases nationally and in Mexico City, respectively. At the national level, but not in Mexico City, PT/SAL followed AMO. OPID had a somewhat similar relative importance at both scales (10.6 and 14.6% nationally and in Mexico City, respectively), but all other intestinal diseases made up strikingly different percentages nationally and in Mexico City. In particular, TF and SHI were ranked higher nationally than in Mexico City, where GIA was more important than at the larger country scale.

As may be expected, the differences in relative importance seen between scales were due to disparities at the level of states and municipalities (Figure 2(b)). Thus, an inspection of the percentage of cases due to particular IID among states or municipalities revealed substantial heterogeneity. This heterogeneity was greater among the diseases with higher number of cases, because there is greater room for variation in those instances.

### 3.3. State and Municipal-Specific Ranking of Intestinal Diseases

**Figure 3:**
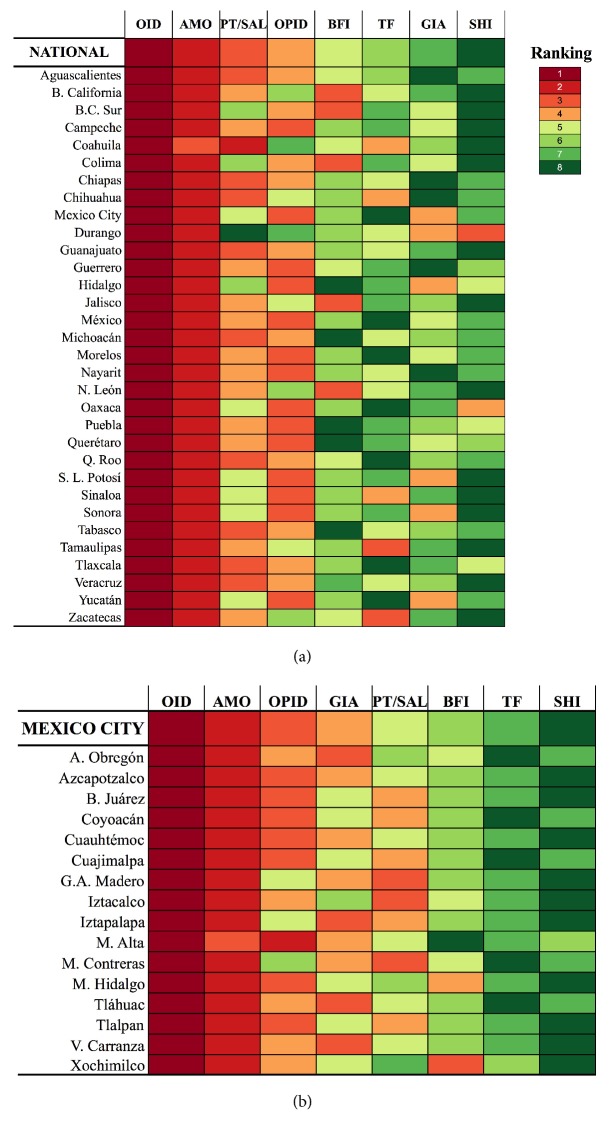
(a) National and (b) subnational ranking of gastrointestinal diseases according to the cumulated number of new cases over the decade.

The national ranking of IID according to the cumulated cases over the decade was OID, AMO, PT/SAL, OPID, BFI, TF, GIA, and SHI (Figure 3(a)). Interestingly, none of the 32 states showed the exact same pattern, and in fact in nine states, including Mexico City, the ranking of IID differed in 6 out of 8 slots from the national pattern. On the other hand, Aguascalientes and Quintana Roo differed in one and two slots respectively from the national ranking, and OID and AMO were ranked 1^st^ and 2^nd^ in 31 of 32 states. BFI, TF, and GIA that make up 3-4% of non-OID cases (Figure 2(a)) appeared shuffled in the ranking of individual states. Interesting “leaps” are observed in Durango, where SHI (8^th^ nationally and in most states) is 3^rd^ in importance; meanwhile PT/SAL, which was the 3^rd^ most important nationally, is ranked in the last place.

Diseases in the municipalities of Azcapotzalco and Cuauhtémoc had the same ranking than in Mexico City: OID, AMO, OPID, GIA, PT/SAL, BFI, TF, and SHI (Figure 3(b)). Despite the heterogeneity in the relative importance of diseases across municipalities, it was not as large as that observed at the national scale: only A. Obregón differed in 6 out of 8 slots of the ranking from Mexico City, and of the three diseases ranked lowest only BFI appeared in third and fourth place in Miguel Hidalgo and Xochimilco. Finally, in half of the municipalities, PT/SAL appeared in third or fourth place but was ranked lower in the other half.

### 3.4. Incidence of Intestinal Diseases in States and Municipalities

The importance of each IID is better appreciated in their median incidences (Supplementary Table 1): nationally OID was the leader of intestinal infections since it caused 5177 cases per 100 000 (95% CI: 4613-5754), followed by AMO, with median incidence of 646.8 (381.2-855.3) cases per 100 000. Three groups of IID showed similar incidence: PT/SAL and OPID, BFI and GIA, and finally, TF and SHI. Among states, the incidence of OID was consistently high whereas GIA and SHI showed the two lowest incidences (Supplementary Table 1).

**Figure 4:**
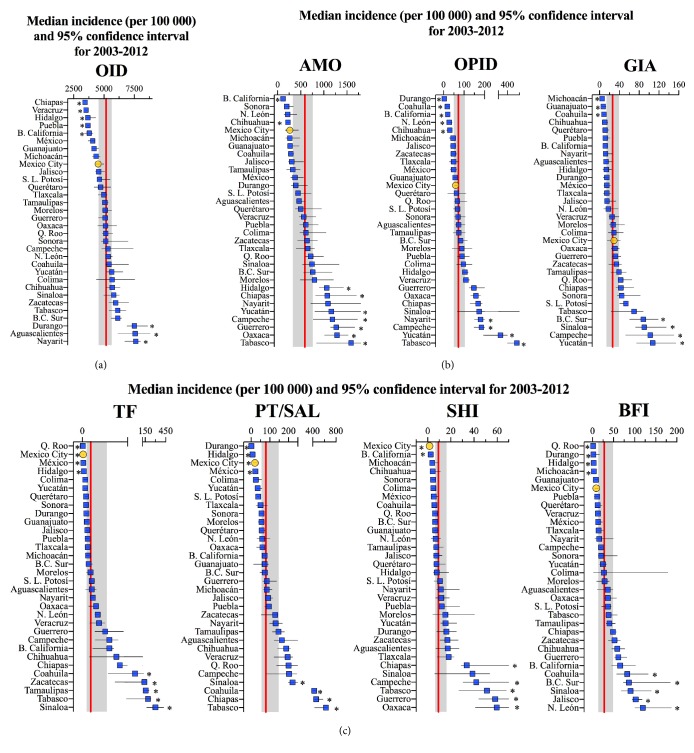
Decade median incidence of IID of (a) unknown origin, (b) due to protozoa, and (c) caused by bacteria in the states of Mexico. Straight line and gray area represent the national median and 95% CI. *∗* indicates significant differences (*p* < 0.05) between state value and national reference.

In Figure 4 we present the decade median incidence in the states compared to the national reference. Mexico City exhibits consistently lower incidence, but this difference is statistically significant only for 3 out of 4 bacterial intestinal infections. States like Tabasco, Sinaloa, and Campeche frequently presented above-median incidence, while in Baja California and Hidalgo the incidence of four disease categories was significantly below the national average. Besides, some states showed contrasting behavior among diseases; for instance, Durango had the third highest incidence in OID but reported low incidence in OPID, PT/SAL, and BFI. Likewise, Coahuila had significantly lower incidence of OPID and GIA than the national median and high values for PT/SAL and BFI. Finally, Aguascalientes had the second highest incidence of OID, but in all other classes its incidence was similar to the national reference.

When analyzing the data with the highest spatial resolution, we found that the overall incidence of IID was significantly below the median for Mexico City in 2 of 16 municipalities (Supplementary Figure 1a). Since OID caused the greatest incidence of all IID (range from 1482 to 9908 cases per 100 000, Supplementary Table 2), the ordering of municipalities in the total incidence followed, with little change, the behavior shown for OID. Tláhuac and Xochimilco were often below the local median incidence, while Azcapotzalco, Cuauhtémoc, and Benito Juárez tended to be above the local median incidence for most individual diseases (Supplementary Figure 1b and Supplementary Table 2).

### 3.5. Geographic Distribution of Gastrointestinal Disease Incidence

**Figure 5:**
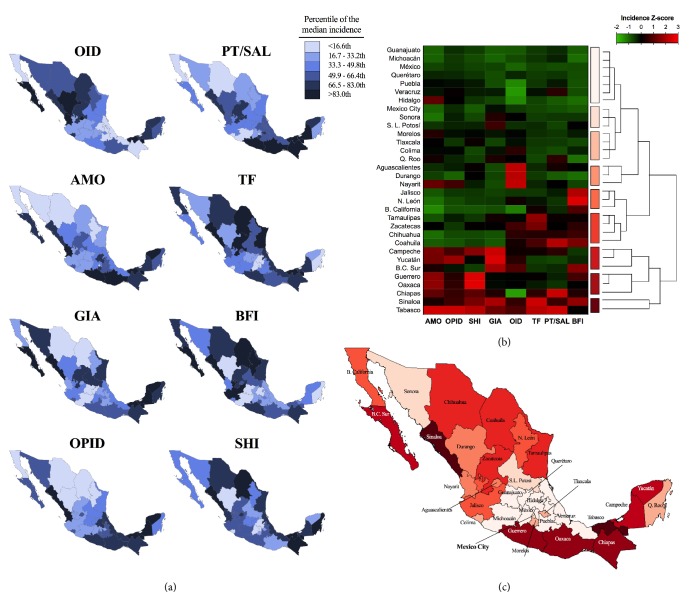
(a) Spatial distribution of the percentile of decade median incidence of each IID for the municipalities of Mexico City and (b) heatmap and cluster analysis of the values of incidence Z-scores.

An alternative illustration of disease incidence by state may be found in the maps of Figure 5, which show the percentile distribution of the decade median. Maps for individual diseases are shown in Figure 5(a), while a multivariate analysis, grouping states by Z-score, is shown in Figure 5(b). Strikingly, the south-southeast and eastern states presented a higher incidence for AMO, SHI, PT/SAL, and OPID, and this resulted in a higher Z-score for the region. On the opposite side of the score, central states (Guanajuato, Michoacán, Estado de México, Querétaro, Puebla, Veracruz, and Hidalgo) all experienced the lowest integrated incidence, and if we consider also the next Z-score ranking (which includes Mexico City and San Luis Potosí), we can identify a low incidence central band in the country.

As depicted in Supplementary Figure 2a, the higher spatial resolution data used in the maps of Mexico City show generally higher incidence of most diseases in Cuauhtémoc and surrounding municipalities, as confirmed by their Z-scores (Supplementary Figure 2b).

### 3.6. Gastrointestinal Disease Incidence and Human Development Index

**Figure 6:**
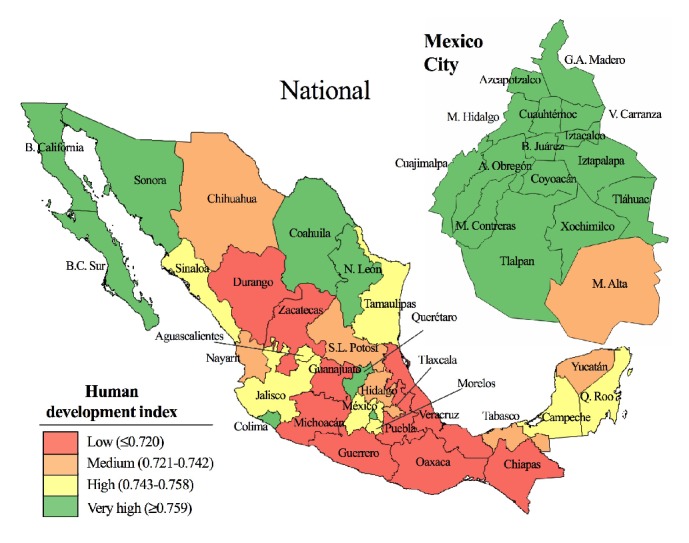
Spatial distribution of Human Development Index (HDI) for the national states and the municipalities of Mexico City.

**Figure 7:**
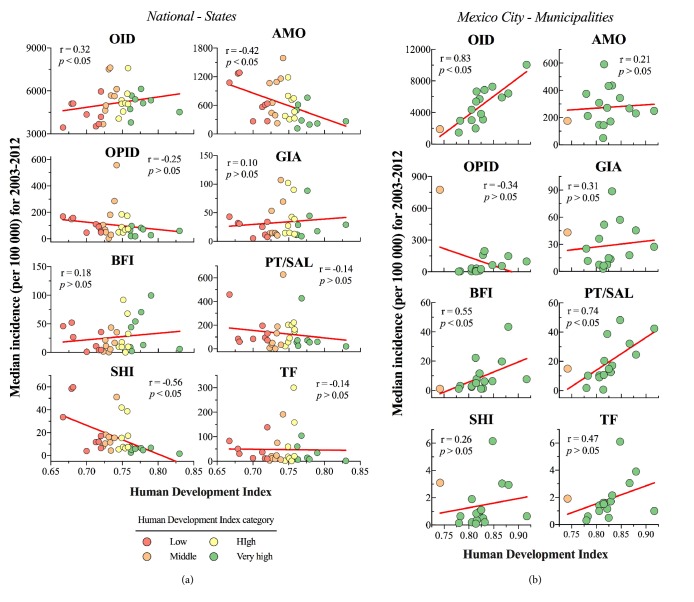
Association of the incidence of intestinal infections and HDI at (b) the state level and (c) the municipal level for Mexico City. The straight lines were drawn from linear regression analysis to assist interpretation of the association.

We measured the relationship between HDI and disease expecting to find negative correlations, i.e., lower HDI, greater incidence. The map depicted in Figure 6 shows the geographic distribution of HDI values; states with low HDI were predominantly located in the Southern and Eastern regions of the country. In sharp contrast, most municipalities in Mexico City had very high HDI. At the national level, only AMO (r = -0.42,* p* < 0.05) and SHI (r = -0.56,* p* < 0.05) were negatively correlated with HDI (Figure 7(a)). Moreover, when looking at the municipalities, no disease class had a significant negative association to HDI. In fact, OID, PT/SAL, and BFI all had significant positive correlations that went from r = 0.55 to 0.83 (Figure 7(b)).

Interestingly, at both levels there was a positive and significant linear relationship between HDI and OID (Figure 7). Notably, Benito Juárez had the greatest HDI (0.917) as well as the greatest median incidence of OID (10049, 95% CI: 9016-10505 cases per 100 000). Whereas, the state of Chiapas with the lowest HDI (0.667) had the 7^th^ lowest median incidence for OID among all regions (3440, 95% CI: 3321-3836 cases per 100 000).

## 4. Discussion

In recent years, six IID were among the twenty most important communicable diseases in Mexico [15], and the results presented herein confirm that IID are of undeniable epidemiological importance both at the national and subnational levels. We describe incidence patterns and relative importance of individual IID among states and municipalities. OID caused >80% of the reported cases at both spatial scales during the decade and thus constitute the main class of gastrointestinal disease. In the national ranking, AMO, PT/SAL, OPID, BFI, TF, GIA, and SHI followed OID. However, as shown by the specific regional patterns found here, any effort aimed to tackle these infectious diseases should be oriented by the specific regional incidence, rather than treated with a single policy. Surprisingly, we found only two significant negative correlations between specific disease incidence and HDI. On the other hand, the analysis of the integrated incidence of all IID in each state indicated that the south-southeast region, which indeed has the lowest HDI values, requires a higher degree of attention and generally greater control and prevention efforts.

Over the last decades there has been a significant reduction in the incidence and mortality of communicable diseases worldwide [1, 3], in conjunction with substantial global and regional decreases in mortality due to diarrhoea [6]. Nonetheless, the highest burden of the gastrointestinal diseases and their characteristic diarrhoea persist as an epidemiological feature of developing countries. Our results confirm this notion, at least for the poorest regions of the country in which we identify a great opportunity for future implementation of region-specific public health policies as well as control and prevention campaigns [42, 43].

The incidence and relative importance of individual IID was found to differ among states and municipalities, but not for OID, which ranked 1^st^ at both scales and caused 52.29 million new cases during the decade. Given that a group of poorly diagnosed diseases (OID) is of paramount importance, it is worth asking whether greater diagnostic efforts could help to reduce the importance of OID by appropriate identification of the etiological agents and tailored treatment that may help prevent further cases. On the other hand, accurate diagnoses often involve lab tests, which most of the time are costlier than the benefit of identifying the cause, so this is an open question.

Even though the epidemiological transition from communicable to noncommunicable diseases may be considered to be in an advantageous stage in Mexico [44], there exists an irregular shift at the subnational level [39]. Our results help understand partly the dissonant subnational (states and municipalities) reduction in IID incidence as a consequence of the heterogeneous incidence and relative importance of each particular disease. To aid this transition, focused interventions may be guided by our cluster analysis of IID incidence. At the state level, Tabasco Oaxaca, Chiapas, Guerrero, Campeche, and Yucatán (all located in the south and southeast part of the country), together with Sinaloa and Nayarit, demand greater control efforts, and these should be concentrated on reducing the incidence of those gastrointestinal diseases causing the highest incidences in such regions, AMO, OPID, GIA, and SHI, and generally improving living standards, including access to clean water and sanitation [9]. Within Mexico City, Cuauhtémoc stands out as having the highest combined incidence reported and is surrounded by municipalities reporting high incidence. Consequently, this region should attract control efforts, but also research into the factors that explain the epidemiological importance of a municipality of such high HDI.

Interestingly, our analyses of the association between gastrointestinal incidence and HDI gave contrasting results at different spatial scales; in Mexico City their linear relations are positive and significant for three diseases, while being negative and statistically significant for two diseases at the state level. This indicates that HDI alone cannot predict IID case reports and leads us to speculate whether other socioeconomic factors may better predict reported incidence at a local level. To approach this, we tested if areas with more developed health infrastructure reported greater incidence, which could reflect an underlying difference in notification rates. Interestingly, we found a significant* negative* relationship between hospital density per 10 000 inhabitants and the total median incidence of IID only at the national level (r = -0.50,* p* < 0.05).

### 4.1. Limitations and Perspectives of Our Study

As any other source of data, there will always be biases, with the degree and type of bias depending on the particularities of the data. Having said that Mexico has a standardized data collection system in place, which monitors 142 diseases of compulsory notification weekly. This should grant certain homogeneity in data quality. Moreover, the national network of clinics and hospitals that forms the basis of epidemiological surveillance is vast, and the available records are representative of the general population. Lastly, this same system will document the actual impact of control efforts, which means that our suggestion to prioritize those states that were ranked as having highest Z-score is informed by, and can be evaluated within, this framework.

Actual epidemiological links between neighboring areas should be studied more carefully through time series analysis of higher temporal resolution. This is of particular importance to determine the existence of epidemiological links between areas of a city. Additionally, if we want to identify the best geographical regions for control strategies, maps for individual diseases can orient public health efforts.

An important limitation from our study is the lack of comparative results with municipalities from other states than Mexico City. Indeed, two main questions arise in this regard: (1) how will the pattern of incidence vary at the municipal level between two states with contrasting HDI values? and (2) how well will the results from the municipalities of Mexico City predict the IID epidemiological landscape of any other municipality with homogeneous socioeconomic status? We thus acknowledge the need to investigate municipal disease-specific patterns of incidence in states with different HDI.

## 5. Conclusion

The results described in the present study emphasize the need to develop local control measures and preventive interventions guided by the incidence of a particular disease rather than socioeconomic indicators alone. Additionally, there is an outstanding heterogeneity in the way a specific disease affects a locality. Therefore, we believe that among the states and municipalities of Mexico City the disease-focused selective model to provide health in people affected by IID should take into account the interrelationship among incidence, geography, and socioeconomic development.

## Figures and Tables

**Table 1:** Definition of the intestinal infectious diseases included in this study according to the International Statistical Classification of Diseases and Related Health Problems 10th Revision (ICD-10) Version: 2016.

**Code**	**Disease**	**Acronym**	**Includes as causal agent**	**Excludes**
A01.0	Typhoid fever	TF	*Salmonella typhi*	

A01.1 to A01.4	Paratyphoid fevers	PT/SAL	*S. paratyphi*	

A02.0 to A02.9	Other salmonella infections		Infection or foodborne intoxication due to any species other than *S. typhi* and *S. paratyphi*	

A03.0 to A03.9	Shigellosis	SHI	*Shigella dysenteriae. S. flexneri, S. boydii, S. sonnei*	

A05.0 to A05.9	Bacterial foodborne intoxications not classified elsewhere	BFI		*Clostridium difficile*, (A04.7); *Escherichia coli*, (A04-0–A04.4); salmonella foodborne intoxication and infection (A02.-)

A06.0 to A06.9	Amoebiasis infection	AMO	*Entamoeba histolytica*	Other protozoal intestinal diseases (A07.-)

A07.0 to A07.9	Other protozoal intestinal diseases	OPID		Giardiasis (A07.1)

A07.1	Giardiasis	GIA	*Giardia lamblia*	

A09.0 to A09.9	Other types of gastroenteritis and colitis of infectious and unspecified origin	OID		Due to bacterial, protozoal, viral and other infectious agents (A00-A08)

## Data Availability

The datasets analyzed in the current study are available from the corresponding author upon reasonable request.

## Supplementary Materials

Supplementary Materials A supplementary material is available as a word document file which contains the following.** Supplementary Table ****1**: median incidence (per 100 000) and 95% confidence interval of intestinal infectious diseases for 2003-2012 at the national level and for the states.** Supplementary Table ****2**: median incidence (per 100 000) and 95% confidence interval of intestinal infectious diseases for 2003-2012 for the municipalities of Mexico City.** Supplementary Figure ****1**: decade median incidence of total cases of IID and intestinal infections of unknown origin, due to protozoa or caused by bacteria in the municipalities of Mexico City.** Supplementary Figure ****2**: spatial distribution of the percentile of decade median incidence of each IID for the states of Mexico and heatmap and cluster analysis of the values of incidence Z-scores.
