# Supplementary material for: Incidence of Intestinal Infectious Diseases due to Protozoa and Bacteria in Mexico: Analysis of National Surveillance Records from 2003 to 2012
Source: Biomed Res Int. 2018 Jul 15;2018:2893012. doi: 10.1155/2018/2893012 (PMC6077666; doi:10.1155/2018/2893012)
Supplement: Supplementary Materials — A supplementary material is available as a word document file which contains the following. Supplementary Table 1: median incidence (per 100 000) and 95% confidence interval of intestinal infectious diseases for 2003-2012 at the national level and for the states. Supplementary Table 2: median incidence (per 100 000) and 95% confidence interval of intestinal infectious diseases for 2003-2012 for the municipalities of Mexico City. Supplementary Figure 1: decade median incidence of total cases of IID and intestinal infections of unknown origin, due to protozoa or caused by bacteria in the municipalities of Mexico City. Supplementary Figure 2: spatial distribution of the percentile of decade median incidence of each IID for the states of Mexico and heatmap and cluster analysis of the values of incidence Z-scores. [file 2893012.f1.docx]

**Supplementary material**

Supplementary Table 1. Median incidence (per 100 000) and 95% confidence interval of intestinal infectious diseases for 2003-2012 at the national level and for the states.

|  | OID | AMO | PT/SAL | OPID | BFI | TF | GIA | SHI |
| --- | --- | --- | --- | --- | --- | --- | --- | --- |
| National | **5,177 (4,613-5,754)** | **646.8 (381.2-855.3)** | **79.1 (54.5-142.6)** | **82.5 (51.5-104.4)** | **27.7 (13.4-48.8)** | **18.4 (9.6-54)** | **39.1 (22.6-54)** | **9.9 (6.9-17.4)** |
| Aguascalientes | 7,586 (6,149-8,805) | 467.7 (415.7-601.8) | 164 (123.6-272.4) | 73.4 (57.1-105.3) | 35.9 (11.4-48.4) | 20.7 (7.2-30.5) | 12.9 (11.1-25.5) | 17.3 (7.2-27.2) |
| B. California | 3,790 (3,390-4,084) | 116.1 (93.9-142.5) | 72.6 (63.8-76.7) | 20.05 (18.3-22.6) | 65.6 (45.5-102.1) | 59.8 (22.7-69.2) | 11.6 (10.1-14.6) | 2.6 (2.4-3.6) |
| B.C. Sur | 6,137 (5,600-6,448) | 760.7 (564.3-1,176) | 73.2 (46.7-80.6) | 82.8 (73.0-115.8) | 86.2 (73.2-184.5) | 14 (9.6-22.3) | 88.35 (60.7-118.5) | 6.4 (4.5-9.2) |
| Campeche | 5,339 (4,833-7,416) | 1,188 (769.8-1,728) | 203.1 (81.4-243.9) | 184.7 (148-207.5) | 20.2 (18.7-21.7) | 59.7 (28.6-78.6) | 102.3 (52.5-150) | 42 (30.6-83.1) |
| Coahuila | 5,412 (5,148-7,033) | 284.4 (228.7-351.8) | 426.6 (373-465.3) | 18.2 (14.1-27.5) | 82.5 (57.4-132.3) | 104 (57.1-124) | 8.9 (6.2-13.1) | 5.8 (4.4-7.6) |
| Colima | 5,677 (4,367-7,541) | 613.8 (480.6-1,046) | 25.5 (16.5-56.3) | 96.9 (64.8-139.1) | 26.8 (2.5-178.9) | 6.6 (2.5-11.3) | 28.7 (17.6-47.9) | 5.1 (2.8-6.5) |
| Chiapas | 3,440 (3,321-3,836) | 1,077 (825.5-1,706) | 458.8 (413-576.2) | 168 (129.8-189.3) | 48.8 (46.9-57.2) | 82.8 (78.7-115.8) | 43.1 (32.8-68.9) | 33.6 (28.9-85.3) |
| Chihuahua | 5,682 (5,180-6,328) | 224.1 (182.3-270.9) | 188.1 (141.8-211) | 29.8 (14.7-37.9) | 58.8 (45.5-76.8) | 75.2 (15.6-96.9) | 10.4 (5.9-16.6) | 4.4 (2.3-11) |
| Mexico City | 4,523 (4,303-4,991) | 265.1 (165.7-450.9) | 20.4 (11.4-35.6) | 59 (53.8-70.9) | 9.1 (6.1-14.2) | 1.9 (1.6-2.4) | 29.05 (15.5-42) | 1.6 (0.9-2.2) |
| Durango | 7,521 (6,907-8,613) | 392.1 (296.9-645.9) | 1.2 (0.5-2.1) | 3.05 (1.4-4.7) | 2.1 (0.4-16.4) | 9.1 (7.1-11.7) | 14.6 (7.3-19.3) | 16.2 (7.8-24.8) |
| Guanajuato | 4,188 (4,001-4,563) | 269.3 (221.9-464.9) | 72.7 (13.3-93.4) | 56.2 (46.6-64.8) | 8.05 (4.6-14) | 10.1 (5-11.9) | 7.6 (4-13) | 6.4 (5.1-9.4) |
| Guerrero | 5,104 (4,555-5,186) | 1,270 (1,146-1,672) | 84 (62.8-136.9) | 148.3 (124-209.6) | 61.6 (52.3-81.4) | 50.7 (28.1-90.9) | 32 (25.4-43) | 58.3 (44.2-82.1) |
| Hidalgo | 3,671 (3,555-4,326) | 1,066 (904.2-1,425) | 7.3 (6.8-9.5) | 103.6 (94.9-119.8) | 2.8 (1.7-4.2) | 3.5 (3-4.6) | 13.6 (6.8-24.5) | 7.9 (5-18.2) |
| Jalisco | 4,557 (4,298-5,065) | 309.1 (231.7-565.3) | 91.05 (79.9-116) | 46.8 (31.9-60.9) | 102.7 (94.8-117.6) | 10.9 (7.6-13.1) | 14.9 (10.1-33.1) | 7.6 (5.1-12.5) |
| México | 4,066 (3,696-4,250) | 377.4 (286-562.7) | 23 (21.1-26.4) | 49.2 (32.4-63.7) | 13.4 (9.9-23.6) | 2.3 (1.6-3.1) | 14.6 (8.9-22.2) | 5.4 (3.5-8.9) |
| Michoacán | 4,348 (4,060-4,673) | 265.6 (178.1-478.1) | 84.2 (67.2-111.9) | 46.4 (26.8-54.5) | 3.2 (1-4.2) | 12.4 (8.3-15.7) | 5.3 (2.8-10.4) | 4.01 (2.7-9.4) |
| Morelos | 5,099 (4,873-5,636) | 798.7 (489.3-1,209) | 56 (38.4-67.7) | 88.7 (42-138.1) | 28.6 (10.1-39.9) | 18.1 (9.2-22.9) | 27.4 (19.7-50.7) | 15.3 (4.5-40.5) |
| Nayarit | 7,621 (6,715-8,023) | 1,092 (717.7-1,819) | 131.2 (99.4-167.3) | 180.7 (149-205.2) | 16.1 (6.1-49.6) | 23.2 (17.2-28.4) | 12.05 (9.2-26.5) | 11.5 (7.8-27.5) |
| N. León | 5,344 (5,158-5,693) | 218.9 (140.1-416.2) | 59.6 (31.9-101.1) | 27.2 (20.6-42.1) | 119.6 (100.7-187) | 34.1 (27.9-37.4) | 17.7 (8.3-37.4) | 6.8 (3-11.1) |

(*Continued*)

Supplementary Table 1. (*Continued*)

|  | OID | AMO | PT/SAL | OPID | BFI | TF | GIA | SHI |
| --- | --- | --- | --- | --- | --- | --- | --- | --- |
| Oaxaca | 5,118 (4,494-6,051) | 1,286 (1,015-1,531) | 60.8 (30.1-74.8) | 157 (132.9-180.1) | 36.4 (27.7-57.1) | 30.3 (14-33.8) | 30.8 (23.4-40.1) | 59.7 (41.2-70.9) |
| Puebla | 3,681 (3,605-3,794) | 611.6 (479.3-880.7) | 94.4 (73.1-106.4) | 89.9 (67.4-126.8) | 11.1 (4.2-14.4) | 11.8 (5.6-14) | 11.5 (8.6-20.8) | 12.1 (10.2-27.8) |
| Querétaro | 4,727 (4,177-5,549) | 500.7 (382.2-951.5) | 56.5 (49.7-75.1) | 62.7 (19.9-109.3) | 12.4 (9.4-22.7) | 8.05 (5.5-12.2) | 11.3 (6.9-18.9) | 7.7 (5.4-15.3) |
| Q. Roo | 5,141 (4,862-5,728) | 716.2 (601.7-994.1) | 200 (136.9-370.1) | 67.4 (52.8-113.3) | 1.6 (0.2-9.4) | 1.2 (0.7-3.6) | 42.7 (37.9-65.3) | 6.3 (4.8-8.8) |
| S. L. Potosí | 4,631 (4,524-5,504) | 442.2 (378.9-729.2) | 38.4 (34.4-41.7) | 67.7 (48.4-76.4) | 36.6 (21.8-44.4) | 20.7 (18.1-27.5) | 53.2 (40.7-57.7) | 10.6 (5.5-13.2) |
| Sinaloa | 5,801 (4,981-6,271) | 747.8 (539.8-1,331) | 222.2 (198-300.4) | 173.8 (67.2-1,803) | 90.4 (68.6-139.6) | 300 (171-421.4) | 90.2 (72.5-134.8) | 38.7 (6.1-53.7) |
| Sonora | 5,142 (4,784-6,981) | 197.2 (178.3-338.2) | 55.5 (44.7-63.4) | 70.8 (52.7-105.5) | 20.3 (13.3-59.2) | 8.3 (4.8-12.1) | 44.7 (35.5-82.2) | 4.7 (3.5-7) |
| Tabasco | 6,125 (5,428-6,818) | 1,592 (843.5-1,961) | 628 (434.1-665.1) | 557.3 (399.7-701) | 38.1 (37.3-59.2) | 191 (97.3-234.5) | 69.6 (23.1-88.1) | 51.1 (27.1-68) |
| Tamaulipas | 5,078 (4918-5,359) | 328.6 (190.9-489) | 146.6 (114-178.6) | 73.7 (47.2-106.3) | 40.05 (32.6-54.1) | 158 (137-207.9) | 39.1 (22.6-54) | 7.4 (4.9-13.7) |
| Tlaxcala | 4,980 (4,523-5,267) | 651.3 (402.5-799.9) | 50.5 (26.6-86.7) | 49.05 (28.1-72.6) | 14.6 (11.7-25.8) | 11.9 (7.4-17.3) | 14.6 (7.2-22.5) | 18.1 (8.1-22.7) |
| Veracruz | 3,518 (3408-3,620) | 566.3 (468.3-826.6) | 195 (123.3-222.8) | 109.2 (95.3-127.5) | 12.9 (11-17.4) | 36.8 (16.5-49.7) | 25.4 (19-39.2) | 11.7 (7-18.8) |
| Yucatán | 5,644 (5,219-6,571) | 1,162 (807.3-2,156) | 35 (30.8-58.2) | 285.4 (194-400.1) | 25.05 (17.2-32.9) | 6.6 (3.8-11.4) | 107.4 (74-154.6) | 15.5 (11.2-25.1) |
| Zacatecas | 5,961 (5,414-7,039) | 637.7 (435.0-865.7) | 129.3 (54-144.3) | 47.8 (38.4-64.9) | 52.4 (37.6-66.2) | 138.7 (72-165.1) | 34.3 (18.6-44.4) | 17.05 (10-26.4) |

Supplementary Table 2. Median incidence (per 100 000) and 95% confidence interval of intestinal infectious diseases for 2003-2012 for the municipalities of Mexico City.

|  | OID | AMO | OPID | GIA | PT/SAL | BFI | TF | SHI |
| --- | --- | --- | --- | --- | --- | --- | --- | --- |
| Mexico City | **4,523 (4,303-4,991)** | **265.1 (165.7-450.9)** | **59 (53.8-70.9)** | **29.05 (15.5-42)** | **20.4 (11.4-35.6)** | **9.1 (6.1-14.2)** | **1.9 (1.6-2.4)** | **1.6 (0.9-2.2)** |
| A. Obregón | 3,037 (2,851-3,366) | 148 (105.2-379.8) | 31.7 (6.6-39.9) | 36.1 (23.9-54.7) | 9.05 (2.1-26.5) | 2.7 (1.1-22.6) | 1 (0-2.6) | 1.9 (1-4.5) |
| Azcapotzalco | 6,864 (6,375-7,003) | 436.1 (222.3-703.4) | 195.7 (104-272.2) | 88.7 (44.9-131.7) | 17.1 (13.7-44.9) | 6.1 (1.7-24.8) | 2.1 (1.2-4.8) | 0.2 (0.2-0.7) |
| B. Juárez | 10,049 (9,016-10,505) | 249.6 (133.1-673.1) | 97.2 (55.8-120.9) | 27.2 (13.5-51.6) | 42.4 (7-111.6) | 7.6 (0.8-13.5) | 1 (0.8-2.3) | 0.6 (0.3-3.9) |
| Coyoacán | 5,907 (4,408-6,452) | 267.8 (130.7-511.3) | 56 (38.8-94.3) | 18.05 (13.5-48.4) | 32.1 (22.4-59.8) | 19.7 (7.1-26.9) | 3.05 (0.8-5) | 3.05 (0.6-5.5) |
| Cuauhtémoc | 7,274 (6,593-9,054) | 344.4 (305.8-562.1) | 63.8 (34.2-204.6) | 57.1 (44.8-98) | 48.2 (32.5-57.4) | 6.3 (1.3-16.2) | 6.1 (3.2-10) | 6.1 (2.1-10.9) |
| Cuajimalpa | 3,827 (3,205-4,966) | 431.9 (274.8-779.9) | 25.5 (10.2-41.9) | 14.8 (10.7-66) | 14.7 (1.6-70.9) | 1.1 (0.5-4.8) | 0.5 (0-1.6) | 1.1 (0-1.6) |
| G. A. Madero | 4,357 (3,429-5,164) | 308.2 (238.4-421.5) | 4.7 (1.1-10.2) | 7.6 (5.7-21.2) | 10.3 (4.6-52.5) | 2.4 (1.3-5.6) | 1.4 (0.5-2.4) | 0.2 (0.1-0.4) |
| Iztacalco | 5,714 (5,073-5,965) | 272.5 (193.9-351.8) | 11.5 (0.8-59.8) | 7.6 (4.4-21.3) | 38.6 (7.5-70.8) | 11.6 (1.3-24.7) | 1.1 (0.3-2.1) | 1 (0-2.3) |
| Iztapalapa | 2,975 (2,561-3,315) | 212.4 (99.1-319.8) | 4.7 (2.1-8.5) | 11.6 (1.7-27.3) | 10.2 (4.5-25) | 3.05 (1.5-6) | 0.6 (0.3-1) | 0.6 (0.2-1.4) |
| M. Alta | 1,898 (1,733-2,850) | 175.9 (117-265.3) | 775.3 (333-1,163) | 43.2 (22.9-65.7) | 14.9 (8.4-35.2) | 1.1 (0-5.4) | 1.9 (0.8-3.8) | 3.1 (0.8-8.4) |
| M. Contreras | 5,397 (4,602-6,284) | 143.7 (67.8-446.4) | 3.7 (0.8-17.2) | 6.5 (2.1-18.8) | 10.7 (2.5-18.8) | 5 (0-18.8) | 1.5 (0-2.9) | 0.8 (0-2.5) |
| M. Hidalgo | 6,421 (6,058-7,289) | 231.1 (147-401.3) | 148.3 (66.8-259.4) | 45.5 (30.6-53.9) | 24.6 (15.8-36.5) | 43.4 (25.5-82.1) | 3.9 (1.1-4.8) | 2.9 (1.3-4.6) |
| Tláhuac | 1,464 (1,292-1,751) | 375.1 (244-735.1) | 2.7 (0.8-7.5) | 25.3 (14.4-40.3) | 1.8 (0.3-6.7) | 1.1 (0-4.7) | 0.3 (0-0.6) | 0.1 (0-1.4) |
| Tlalpan | 3,120 (2,641-3,521) | 171.8 (68.7-258.4) | 158.2 (111-171.1) | 13.7 (5.5-26.4) | 12.6 (2.9-36.4) | 1.3 (0.5-2.6) | 1.7 (0.6-2.5) | 0.5 (0-0.9) |
| V. Carranza | 6,665 (6,163-7,053) | 591 (265- 836.4) | 31.5 (21.1-53.8) | 51.7 (25.1-146) | 20.4 (15.3-44.8) | 7.7 (3.9-16.7) | 1.6 (0.5-3) | 0.3 (0.2-1.6) |
| Xochimilco | 1,998 (1,770-2,148) | 49.4 (25.8-100.5) | 8.9 (1-21.9) | 3.05 (1.4-11.6) | 0.6 (0.2-1.9) | 22.2 (11-28.4) | 1.6 (0.2-5.8) | 0.1 (0-1) |

Supplementary Figure 1. Decade median incidence of (a) Total cases of IID, and (b) Intestinal infections of unknown origin, due to protozoa or caused by bacteria in the municipalities of Mexico City. Straight line and gray area represent the median for Mexico City and its 95% CI. * Indicates significant differences (*p* < 0.05) between municipal value and Mexico City reference.

Supplementary Figure 2. (a) Spatial distribution of the percentile of decade median incidence of each IID for the states of Mexico, and (b) Heatmap and cluster analysis of the values of incidence Z-scores.
